# Supplementary material for: Are carbon emissions trading and green financial instruments synergistic? -Comprehensive quantitative research based on content analysis
Source: PLoS One. 2024 Mar 7;19(3):e0298601. doi: 10.1371/journal.pone.0298601 (PMC10919601; doi:10.1371/journal.pone.0298601)
Supplement: S1 Data — (ZIP) [file pone.0298601.s001.zip › Data/地级市Z_F工作报告.zip!/┴╔─■/╕o╦│╩╨/└┤╫╘╣1⁄2╓┌║┼í╛┬φ┐╦╩2╛▌═°í┐_2006.docx]

各位代表：

　　现在，我代表市人民政府向大会报告工作，请予审议，并请政协各位委员和其他列席人员提出意见。

　　 一、2005年主要工作回顾

　　 2005年是“十五”计划的最后一年，也是抚顺发展史上很不平凡的一年。面对发展改革稳定的艰巨任务，面对突如其来的特大洪涝灾害，面对原油、原材料价格大幅上扬等不利因素，我们在市委的正确领导下，在市人大和市政协的监督支持下，上下同心，开拓进取，合力攻坚，扎实工作，实现了经济社会的协调快速发展，基本完成了市十三届人大三次会议确定的任务，较好地完成了“十五”计划确定的各项目标。预计全年完成地区生产总值420亿元，同比增长13.5%，五年年均递增12.3%；其中第一产业增长3.9%，第二产业增长14.8%，第三产业增长13.5%。完成固定资产投资150亿元，增长44.4%，年均递增28.2%。社会消费品零售总额177.8亿元，增长12.9%，年均递增9.7%。全地区财政一般预算收入22.3亿元，增长22.2%，年均递增11.3%;一般预算支出48.8亿元，增长23.8%，年均递增13.7%。城市居民人均可支配收入和农民人均纯收入分别为8000元和3740元，增长14.2%和4.5%，年均递增9.2%和5.2%。

　　 国民经济总体发展势头良好。一是工业经济增长内在动力增强。投资74亿元建设重点项目100项、竣工36项，新增销售收入超亿元的企业10户，规模以上工业预计完成增加值140亿元，同比增长14.8%；抚电两台30万千瓦热电联供机组、东露天能源综合开发项目已经国家核准，特别是百万吨乙烯等一批重大项目前期工作进展顺利。二是农业和农村经济稳步发展。围绕绿色特色农产品深加工和农民增收，大力推进农业产业化经营，农业产业化收入占农民总收入的46.2%；投资3.9亿元，建设了关山水库配套等一批水利工程项目；植树造林17.8万亩,封山育林30.8万亩。三是第三产业发展迅速。成功举办了“满族风情国际旅游节”，平顶山惨案遗址纪念馆和战犯管理所被列入全国百家红色经典旅游区，清前史迹游、自然生态游、红色经典游渐成规模，旅游总收入增长37.6%；新型商业业态迅速发展，全市商业网点已达3.9万个、从业人员20.8万人；房地产开发平稳推进，竣工面积增长12.5%；信息产业增势强劲，销售收入、增加值分别增长21%和25%。四是以民营经济为主体的县区经济快速发展。新增规模以上民营企业100户、销售收入超5000万元的20户，民营企业完成增加值180亿元、同比增长25.2%，6个县区、抚顺经济开发区财政一般预算收入超亿元，实现了历史性突破。

　　 改革开放取得明显进展。按照“2005年攻坚、2006年收尾”的目标，共完成国企改制180户，是前3年的总和，特别是新抚钢等13户规模较大的国企改制取得突破，可吸引外来投资72.2亿元，预计改制后销售收入和税收可分别增长3.5倍和7.2倍。对外开放取得新进展，招商引资呈现上升态势，一批利用外资大项目相继落户抚顺，其中STX跨国公司大幅增资、皇家极地海洋世界项目和BSA集团收购顺华铝轮毂，标志着我市外资企业增资、现代服务业和国有企业利用外资的三大突破。全年引进域外资金47.5亿元，增长76.6%；实际直接利用外资4200万美元，增长28.9%；外贸出口3.9亿美元。

　　 城乡面貌进一步改观。坚持用科学发展观指导城市建设，全年改扩建城区道路145条、113万平方米，其中东西四路商业街改造仅用60天，永安桥重建仅用189天，创造了抚顺建设史上设计优、投资省、质量高的典范，深受市民好评；更新“三供”管网22.6公里、排水管网24公里；整治浑河城市段干流和支流堤坝近10公里。加强城市综合整治和违章建筑清理，实现10条街路的占路市场退路进厅，完成60个住宅小区、109万平方米的环境建设；城区植树103万株，新增绿地100公顷。取缔1吨以下直燃原煤锅炉，投入9000万元完成望花地区10个大气污染治理项目，全年城区空气质量达到二级以上标准比上年增加15天。交通基础设施建设完成投资14亿元，新建黑色路面538公里，抚沈高速公路路基和桥涵工程全面完成，新增通油路村111个，通油路村已达75.7%；完成抚顺通往铁岭、本溪、沈阳三大出口建设任务。碧水蓝天、顺畅快捷正在逐步成为抚顺的现实。

　　 为群众办实事力度加大。棚户区改造实现历史性突破，共拆迁集中连片棚户区15片、125万平方米，涉及群众近13万人，拆迁规模为启动棚户区改造18年来总和的1.69倍，建设回迁安置用房188.5万平方米，其中一期60万平方米竣工并于去年11月中旬陆续配户，3.7万人喜迁新居，圆了棚户区几代人的梦，创造了“抚顺速度”和“棚改精神”。采沉区居民安置小区21.8万平方米住宅交付使用，3345户居民将搬入新居。解决吃水难1.3万户、煤气拖期开栓1362户。社会保险扩面完成省下达计划，离退休人员养老金、失业人员救济金保证了100%按时足额社会化发放。提高了城市低保标准，保障人数由16.4万人扩大到17.4万人；筹措款物2600万元，救助城乡临困群众23万人次。完成就业培训2.6万人、创业培训900人、农村劳动力转移培训2.4万人，6910户零就业家庭基本实现了至少一人就业，全市实现实名制就业再就业12.7万人次，其中劳务输出3.6万人次。社区建设得到加强，125个社区办公用房由60平方米以下扩大到100平方米以上，社区办公经费、社区工作者补贴分别提高66.6%和21.6%。

　　 社会大局基本稳定。有效整合资源，在全省率先实现“110、120、122和119”四台合一，完成了城市应急救援接处警中心建设，提高了应急救援的快速反应能力，刑事犯罪发案同比下降4.6%，群众的安全感有所增强。严格信访责任制，加大下访力度，实行标本兼治，集中力量解决重点疑难信访案件，进京、去省集体访分别下降41.7%和30.4%。真心实意为群众排忧解难，市长公开电话转办问题解决率达到91.8%。争取央行专项贷款10.3亿元，解决了遗留12年之久的重点工程债券问题，维护了金融秩序稳定。

　　 抗洪救灾取得重大胜利。面对历史罕见的特大洪水，各级党政组织带领群众奋起抗灾，各县区领导干部不顾危险，坚持一线指挥，共转移遇险群众10万人、解救4000人，将损失降到了最低限度。在救灾重建过程中，广大干部群众积极响应号召，社会各界纷纷慷慨解囊，多方筹集并及时下摆资金5430万元，确保了灾民基本生活，确保了受灾学校按时开学，确保了水毁房屋群众在入冬前搬入新居，实现了大灾之年无大疫，水毁的交通、通信、电力、水利等基础设施基本恢复，灾区生产生活稳定。在特大洪灾面前，我们经受住了考验，极大地鼓舞了全市人民战胜困难的信心和勇气，为抚顺振兴又增添了一笔精神财富！

　　 社会事业协调发展。积极开发人才资源和引进国外智力，增强科技对经济的支撑作用，推动企业与境外、域外科研院所加强技术合作与交流，实现高新技术产业增加值36亿元，增长30.8%。教育布局调整取得实质性进展，撤并中小学45所、中等职业技术学校7所，改造农村D级危房学校40所、2.6万平方米，抚顺职业技术学院与辽宁石油化工大学联合办学，实现了市属高校本科专业零的突破。群众文化活动蓬勃开展，广场文化演出2000余场、观众达150万人次，大型话剧《赵景顺》在全省巡回演出获得巨大成功，“满族秧歌”被列为首批省级非物质文化遗产保护名录第一位。公共卫生体系不断完善，防治重大传染病取得显著成效，农村新型合作医疗试点进展顺利，社区卫生服务覆盖城市人口达到98%；建筑面积达2万平方米的中心医院现代化门诊大楼，仅用半年时间就建成投入使用。群众性体育活动日趋活跃，竞技体育水平有所提高，夺得4枚全运会金牌。广播电视节目质量明显改进，实现农村有线电视村村通。继续保持低生育水平，人口素质有新提高。

　　 政府自身建设进一步加强。依法向市人大及其常委会报告工作并认真执行其各项决议，定期向市政协通报工作并主动接受其民主监督，全年共办理人大代表建议334件、政协提案624件，办理结果满意率达99.3%。完成新一轮政府机构改革，实施工作目标责任制，公务员队伍管理得到加强。全面完成乡镇事业单位改革，启动了市属事业单位改革。公共行政服务中心正式投入使用，进入中心办理的行政审批事项316个、便民服务项目14个，累计接待群众10万多人次，受理审批及服务事项5.6万件，办件速度比承诺时限平均提前3.6个工作日。强化绩效考核力度，机关作风和工作效率有所提高，地区发展软环境进一步改善。 各位代表，回顾一年来走过的路，我们没有急功近利，而是着力解决事关长远发展的根本问题，为抚顺今后的大发展奠定了坚实基础；面对困难，我们坚信事在人为，办法总比困难多，棚户区改造、抗洪救灾等突破性的工作，再次证明了抚顺人能吃苦，能干事，能创造奇迹。在此，我代表市政府，向为抚顺改革开放和加快老工业基地振兴付出辛勤劳动的全市人民，向给予政府工作大力支持的人大代表、政协委员和各民主党派、工商联、无党派人士、群团组织、离退休老同志，向为抚顺发展建设做出积极贡献的中省直部门、驻抚部队、武警官兵、政法干警，向所有关心和支持抚顺发展的各界人士、海内外朋友，表示衷心的感谢并致以崇高的敬意！

　　 在看到成绩的同时，我们也清醒地认识到经济社会发展中存在的问题。主要表现在：一是经济总量小，发展速度和质量有待进一步提高，财政包袱沉重。二是结构调整任务艰巨，改革难度大，对外开放程度低。三是社会保障压力大，就业矛盾比较突出，还有近8万人没有实现相对稳定就业。四是少数干部责任感不强，作风不实，办事拖拉、推诿扯皮现象时有发生，发展软环境还存在不少问题。五是一些关系群众切身利益的问题尚未得到根本解决，群众吃水难、如厕难、供暖差、安全感不强等问题仍然存在，城市总体管理水平还不高。针对这些问题，我们已经并将继续采取有效措施，认真加以解决。

　　 二、“十一五”发展目标和主要任务

　　 今后五年是抚顺实现振兴的关键时期，我们面临许多难得机遇和有利条件。从外部环境看，世界经济正处于新一轮的上升期，国内经济处于扩张阶段，国家继续支持东北等老工业基地振兴，“南资北移”势头强劲，有利于我们吸引外来资金、调整和提升经济结构。从抚顺自身看：一是招商引资条件优越。水、土地和劳动力等要素资源丰富，人均水资源量高于全省平均水平40%；通过棚户区改造和采煤沉陷区治理，加上闲置存量，可用土地达3400公顷；产业技术工人资源丰富。二是制约发展的障碍性因素逐步得到解决。国企改制即将全面完成，加速发展的动力机制基本建立；矿区转产转型步伐加快，一些项目成为全市新的经济增长点；城市基础设施进一步完善，综合整治力度加大，软硬环境逐步改善，有利于我们吸引外资、加速发展。三是中省直企业和地方重点转制企业改造扩能投资大、后劲足。中油抚顺石化公司100万吨乙烯、辽电和抚电、新钢铁改造等一批规模大的企业和项目将陆续启动，目前可预见的技改扩能投入就可达数百亿元，新增销售收入上千亿元。四是上下一心求发展的愿望十分强烈。我们拥有一支忠于职守、勇于创新、真抓实干的干部队伍，特别是通过学沈阳、找差距，全市上下求发展、务振兴的积极性空前高涨。

　　 基于上述分析，我们制定了“十一五”实现跨越式发展的目标，即到2010年，地区生产总值年均增长16%以上；固定资产投资累计完成1500亿元，比“十五”时期增长2.3倍；全地区财政一般预算收入50亿元，年均增长17.5%；城市居民人均可支配收入和农民人均纯收入年均分别增长11.8%和11.7%。万元生产总值能耗降低20%。

　　 我们有理由相信，今后五年，将是抚顺历史上发展最快的五年，抚顺人奋发有为的五年，人民群众得到实惠最多的五年！

　　 实现“十一五”奋斗目标，我们要把握全局，突出以下工作重点：

　　 1、统筹发展，提升地区经济整体实力。在抓好老城区改造，特别是中部商业中心区改造的同时，实施“西进、北拓、南治、东优”，扩展城市发展空间，优化城市发展布局。进一步强化对农业的支持，推动农业结构调整，把抚顺建成具有特色优势的农产品生产、加工基地。到2010年，农产品深加工率达到35%以上，50%的农村劳动力从事非农产业，农民收入70%以上来自农业产业化，全面提高农村的生产水平和农民的生活水平。优化工业结构，转变增长方式，大力发展石化、冶金、装备制造等优势行业和优势企业，强力推进并形成石化、钢铝、油母页岩、山区资源4大精深加工体系，重点发展抚顺经济开发区，建设高新技术、钢铝加工、塑料加工、机械加工、石蜡加工5个产业园区，实现资源型城市向资源深加工型城市的转变。到2010年，石化工业销售收入达到700亿元，以“两钢一铝”为主的冶金业达到400亿元，装备制造业达到150亿元，高新区等5个工业园区达到570亿元，资源深加工产业达到460亿元。推进服务业发展，全面提高社会化服务水平，重点发展潜力较大的旅游业和商贸流通业。到2010年，旅游业总收入52亿元，消费品零售总额350亿元，年均分别增长23%和14.5%。

　　 2、扩大开放，推进抚顺振兴。突出对外开放的龙头作用，以开放促国企改革、体制创新，促结构优化和调整，促资源型城市转型，促经济快速发展；加大招商引资力度，改进方式，拓宽渠道，提高利用外资的质量和水平；鼓励国有大中型企业和民营企业走出去，以投资办厂和境外上市等方式，积极利用国外的资金、市场、资源、人才，在更加广阔的空间实现更大发展。五年累计吸引国内外资金800亿元，比“十五”增长3.5倍，其中实际利用外资的比重达到10%以上。

　　 3、深化改革，加快体制机制创新。不断深化企业改革，加速建立现代企业制度，提高企业核心竞争力；加快行政管理体制和事业单位改革，转变政府职能，切实把政府经济管理的着力点，转移到为各类市场主体服务和创造良好的发展环境上来；积极推进财税、金融和农村经济体制改革，使各项改革相互促进，推动体制创新，促进抚顺全面振兴。

　　 4、优化环境，增强地区整体竞争力。围绕塑造城市新形象，按照现代化标准规划、建设和管理城市，广泛吸纳社会资金参与城市建设，加大环保和绿化力度，加快基础设施建设和改造，构筑现代交通运输体系，使抚顺更加适合人居，有更强的对外吸引力。到2010年，公路总里程达4600公里。建设抚顺环城高速公路和环城快速干道，续建抚沈高速公路，新建南杂木至通化、南杂木至吉林、辽宁中环线、永陵至桓仁、三宝屯至石文高速公路，实现村村通油路、县县通高速。加强城市供水、供燃气、供暖和垃圾处理、污水处理系统建设。改善浑河流域生态环境，加快矿山生态环境保护和整治，搞好城市森林建设，实现“林在城中、人在林中”的城市生态环境目标。全市森林面积达到1190万亩、林木蓄积量6400万立方米；“一区一坑三场”土地整理面积4000公顷以上。树立软环境就是生产力和竞争力的观念，认真贯彻《抚顺市优化经济发展环境条例》，整合资源、精简审批、优化流程、公正执法，提高工作效率和质量，使地区投资环境得到明显改善。

　　 5、以人为本，提高人民生活水平。坚持把改善城乡人民生活作为一切工作的出发点和落脚点，从群众最需要的地方做起，从群众最不满意的地方改起。提高全社会就业水平，尽快完善养老、失业、医疗和低保等社会保障体系，加大对特困群体的帮扶力度，尽最大可能解决群众关心的热点和难点问题，确保群众安居乐业。大力发展教育、文化、卫生、体育等各项社会事业，让人民群众真正享受到改革开放的成果。“十一五”时期，全市城镇实名制就业达到35万人以上。到2010年，全面完成棚户区改造和采煤沉陷区居民搬迁，完善棚户区的公用设施和生活配套设施，实施居民小区地表土无裸露计划，90%的社区达到市级和谐社区标准，100个社区进入省级标准化社区，全市进入国家平安市行列。

　　 三、2006年重点工作

　　 2006年是实施“十一五”规划的第一年。今年政府工作的指导思想是：以邓小平理论、“三个代表”重要思想和党的十六届五中全会精神为指导，紧紧抓住国家振兴东北等老工业基地的历史机遇，进一步深化改革、扩大开放、调整结构、优化环境，以项目为重点，以工业园区为载体，加快资源型城市向资源深加工型城市转变，推进社会主义新农村建设，促进经济社会全面协调可持续发展，努力建设资源节约型、环境友好型、社会和谐型的新抚顺。

　　 2006年全市经济社会发展的主要预期目标是：地区生产总值增长15%以上；全社会固定资产投资完成200亿元，增长33%；实际直接利用外资1亿美元，增长138%；全地区财政一般预算收入24.4亿元，增长11.9%；当年财力口径支出34.5亿元，增长15.5%；城市居民人均可支配收入和农民人均纯收入分别增长12%和10%。万元生产总值能耗降低5%。

　　为实现上述目标，2006年我们要突出抓好九个方面的重点工作。

　　 (一)全面提高对外开放水平，促进地区经济快速发展

　　 市委、市政府将今年确定为“开放年”，就是要在全市形成上下一致想开放、方方面面促招商的浓厚氛围，并将对外开放贯穿到各项工作中去，以开放推动经济的快速发展。

　　 转变招商方式。树立大开放观，坚持“域内、域外、境外”三资并重，实行全方位、宽领域、多渠道的对外开放，鼓励外资参与国企转制，进入公用事业、农业、城市基础设施等领域。建立政府主导、企业主体、园区主战场的招商机制，特别是要启动建设东洲高新技术产业园、台湾工业园等园区建设，为招商引资提供载体。确定重点招商国家和地区、目标投资商和知名企业，以韩国、马来西亚、日本、新加坡和台湾、香港及长三角、珠三角等资本密集地区作为重点，采取代理招商、以商招商等有效形式，实行小团组、点对点、户对户的接触、洽谈，主动出击，以重点突破带动全局推进。培育出口产品加工区，完成外贸出口额5亿美元，增长28%。

　　 加强项目策划。以市场为导向，围绕国家产业政策和支持方向，编制《抚顺市投资优势产业指导目录》，策划包装一批300至500亿元规模的项目充实到项目储备库，做好招商准备。围绕比较优势和产业发展方向，在严格环保准入的前提下，重点引进一批投资规模大、科技含量高、拉动力强的龙头项目，吸引配套企业跟进，全年引进境外投资500万美元以上的项目10个、国内投资超亿元的项目25个以上。抚顺经济开发区要切实发挥先导示范作用，全年实际直接利用外资3000万美元，增长25%。

　　 健全激励机制。将全市对外开放的各项指标层层分解，落实到县区、部门和企业，明确任务和责任。坚持定期调度，加强督促检查，定期通报各单位的指标进度情况。全面落实项目责任制，对落地项目实行“一条龙”服务，并严格考核和奖惩，把上项目、吸引投资作为县区及相关部门年度考核的重要内容，形成在投资上比高低、以项目论英雄的氛围和机制。 进一步改善投资环境。完善并灵活运用政策，重点支持前景好的项目。下放外商投资项目委托审批权，转变政府职能，创造公正透明的法制环境、长期稳定的政策环境、全方位的服务环境、最具吸引力的人才环境和高品位的人居环境。树立“养商重于招商”理念，全面推行首问负责制和检查企业登记卡制度。组建市县（区）两级“外商投资企业协调服务中心”，努力为外商投资企业排忧解难。

　　 (二)转变增长方式，做强做大工业经济

　　 抚顺经济的基础和支撑是工业，潜力和优势也在工业。全年要实施千万元以上工业项目150项，投资突破100亿元，增长25%；新增规模以上工业企业150户以上，规模以上工业增加值增长18%以上。

　　 发展壮大石化产业。支持中油抚顺石化公司实施“千万吨炼油、百万吨乙烯”战略，建设800万吨蒸馏装置、60万吨酮苯脱蜡等项目，确保100万吨乙烯改造工程开工；支持中油抚顺石油化工公司搞好聚丙烯催化剂、9.6万吨丙烯腈装置扩建等项目建设；鼓励和支持中省直企业与地方经济融合，加强协作配套，实现共进互赢。

　　 改造提升传统产业。鼓励各类企业用高新技术和先进适用技术加强技术改造，扩大生产规模，淘汰工艺落后、污染严重的粗加工项目，提升传统产业的经济结构和生机活力。支持抚矿集团加快战略转型，大力发展接续产业和新兴产业；支持新钢铁技术改造、抚顺发电厂两台30万千瓦热电联供机组等重大项目建设。完成技改投入75亿元，增长25%。

　　 加快工业园区建设。按照市管规划、区管投资建设和招商的原则，启动东洲高新技术产业园、新抚塑料深加工、望花钢铝深加工、顺城石蜡深加工和机械制造等5个工业园区建设，完成基础设施建设投入2.4亿元，确保一批市场前景好、创新能力强的企业进驻。

　　增强企业核心竞争力。建立健全激励创新政策，构筑以企业为主体的技术研发体系，加强与科研院所合作，提高企业消化吸收再创新、继承创新和原始创新能力；引导生产要素向名牌产品和优势企业集中，争创名牌产品；完善激励机制，加强企业家队伍建设。

　　（三）深化各项改革，增强地区发展的动力和活力

　　 完成国企改制任务。以资产盘活、企业发展为目标，积极引进战略投资者，集中各种资源，加大支持力度，确保改制企业具有较强的成长性。按照因企制宜、一厂一策的原则，用好用足政策，依法规范操作，维护职工权益，在保证质量的前提下全面完成国企改革。加快事业单位改革。坚持积极稳妥、加速推进的原则，搞好事业单位分类改革，经营性事业单位要全部转为企业。全面推行聘用制，搞活内部分配机制，精简机构和人员，务求事业单位改革取得明显成效，解决政事不分、效率低下的问题，增强事业单位发展活力。

　　 深化农村各项改革。围绕促进农民增收和农村经济发展，推进产业向优势区域集中、耕地向种植能手集中、养殖向小区集中、加工向园区集中、农民向城镇和大村集中。加快农村集体林改革。加强农村集体经济财务管理。

　　推进财政管理体制改革。全面实行部门综合预算和国库集中收付制度，严格收支两条线管理，强化收缴分离，完善并严格执行政府采购制度，加强财政和审计监督，提高财政资金的使用效率和效益。树立“过紧日子”的思想，按照公共财政的要求，调整优化支出结构，强化预算约束。加强诚信体系建设，构建银企合作平台，加大金融支持地区经济发展的力度。

　　（四）以促进农民增收为核心，加快社会主义新农村建设

　　 发展特色产业。坚持用工业理念谋划农业，构建粮、畜、禽、菜、药、果等六大深加工产业集群，突出“特色”和“效益”，绿色、无公害和有机农业的规模扩大到100万亩，受益农民10万户；扶持重点产业化龙头项目45个，受益农民14万户；大力发展林地经济，开发林下资源13万亩，新增林业总产值4亿元。发展壮大农村经济合作组织，提高农民组织化程度和农产品市场竞争力。加强畜禽防疫体系建设，有效防控疫情发生。

　　 加强基础设施建设。水毁公路9月底前恢复灾前水平，水毁水利设施基本恢复，重点河流险段在搞好规划的基础上得到全面防护。抚沈高速公路竣工并投入使用；新增黑色路面100公里，旅游环路全线竣工。农村自来水普及率达到64%，新建水利设施300处。通信实现村村通。

　　 改善农村环境。坚持积极推进、试点先行，加强村镇统一规划，按照“生产发展、生活富裕、乡风文明、村容整洁、管理民主”的目标，抓好50个市级和一批县级新农村建设试点，引导农村走向更加富裕和文明之路。完成植树造林8万亩、封山育林30万亩，新建沼气大棚4650座，推广大型沼气池。

　　 加快县城和小城镇建设。加强指导扶持，注重发挥特色，在统一规划的基础上，突出抓好新宾镇、清原镇两个县城和南杂木、永陵、大四平、草市、红透山、石文、后安等小城镇建设，增强其吸引力和辐射力。加大扶贫开发力度，全年减少贫困人口2.5万人。

　　 （五）大力发展县区经济、民营经济，壮大提升第三产业

　　 加快发展县区经济。高度重视县区发展，着眼于县区尽快做强壮大，建立激励县区发展的长效机制。全面实行统一规范的分税制财税体制，市本级除保留7个门类的典型企业外，其余所有企业全部下放实行属地化纳税，在保既得利益的基础上，建立市与县区利益共享、风险共担的激励机制，规范税收秩序，调动县区加快发展的积极性。真正还权和放权于县区与基层，强化县区综合管理职能。采取更加灵活的政策，支持和鼓励县区培育发展特色优势产业群。全年县区固定资产投资增长50%，生产总值和财政收入分别增长24%以上。

　　 促进民营经济大发展。鼓励全民创业，使更多的人通过诚实劳动和合法经营，成为经营者和企业家。支持民营经济以多种形式参与国有企业、集体企业转制和进入公用事业领域。以建立融资担保体系为重点，加大扶持力度，切实解决制约民营经济发展的瓶颈问题。引导民营企业加快股份制改造，积极引进新技术、新工艺和新产品，实现机制管理创新和做强做大。全年完成民营企业增加值225亿元、上缴税金15亿元，分别增长25%和20%；培育销售收入超亿元企业10户以上。

　　大力发展第三产业。旅游业要打好满清文化游、山水特色游和红色旅游等品牌，扩大抚顺影响力；以沈阳“世园会”为契机，加快旅游产品的开发推介和基础设施的建设完善，提高接待能力，办好2006中国（抚顺）满族风情国际旅游节，来抚游客、旅游收入分别增长15.4%和12%。商贸流通业要围绕提高档次和水平，加快发展物流业，积极引进知名商家，按照现代化标准改造、建设和整治站前商业区，完善裕民市场群，鼓励商贸流通业向社区和农村延伸。房地产业要大力开发面向群众需求、适销对路的住房，全年施工面积164万平方米，竣工面积115万平方米。信息产业要加快发展，全年实现增加值18.5亿元，增长22%。

　　(六)加强基础设施建设与土地整合利用，提升城市功能形象科学谋划城市

　　按照打造现代工业城市、突出“山水”特色城市的思路，有效整合优化土地资源，高水平规划、设计和建设城市，形成以浑河景观带为轴的中部人居环境优良区，以工业园区为主体的工业集聚区，提高城市的功能形象和对外吸引力。

　　 高质量建设城市。在多方筹资、合理负债的基础上，加大城市建设力度，优先“雪中送炭”项目，增强精品意识，提高城建项目质量。启动“两园一带一馆”，即劳动公园与高尔山公园、浑河景观带重要节点、平顶山惨案遗址纪念馆的改造和建设。改造道路128条、75公里、90万平方米，重点改造建设“四条路”，即改造南环路、高山路，东延临江路和新城路。实行“三扩”，即扩建将军水厂、和平水厂和吴家堡水厂。改造建设地下管网100公里。整治“两区一口”，即站前商业区、居民小区和城市西出口周边环境。打通“三个通道”，即前甸通道和戈布通道分别与抚沈高速相连、高阳通道与沈通线相连。

　　 强化城市管理和土地经营。理顺执法体制，切实解决多头、缺位执法和扯皮问题；按照权责统一的原则下放权限，发挥城区作为城市管理的主体作用；强化市容市貌整治，下力气解决“五乱”问题，实现10条街路占路市场退路进厅，确保城市秩序和面貌大改观。增强市场化理念，结合企业改制、棚户区改造和采沉区整治，加大土地整理储备力度，实行净地出让，全年收购土地300公顷，招、拍、挂出让200公顷，分别增长1倍以上。

　　 加大环境综合整治力度。一要管好天，确保城区空气环境质量达到或好于二级以上标准的天数不少于280天，望花地区空气环境质量达到或好于三级标准的天数不少于330天，实现空气清新。二要管好水，浑河市区段水质达到五类水体标准，实现河流清澈。三要管好生活环境，加强垃圾和污水处理，垃圾无害化日处理增长50%，污水处理率提高30%；市区植树100万株，新增绿地50公顷，实现, 环境清洁。四要管好矿业准入，强化矿山开采监管，加强矿山生态恢复和整治，保护绿水青山。

　　(七)坚持以人为本，加快构建和谐抚顺推进棚户区改造

　　继续把棚户区改造作为“一号民心工程”，确保完成列入省计划的150万平方米集中连片棚户区拆迁改造任务，对未列入省计划的棚户区也要积极争取加大拆迁改造力度，让所有棚户区居民都能尽早圆上住楼梦。坚持棚户区改造与完善城市基础设施、建设功能新区和发展劳动密集型企业相结合，确保回迁居民搬得进、住得稳、环境好。

　　 加强社会保障和再就业工作。强化社会保障体系建设，依法加大社保基金征缴和清欠力度，扩大养老、失业、医疗和工伤保险覆盖面，养老保险覆盖面达到90%以上；加强城市低保动态管理，确保符合条件的困难居民应保尽保；完善扶贫救助体系，保证失地农民、受灾居民和贫困家庭等弱势群体的基本生活；启动并推进农村社会保障体系建设。认真落实各项扶持政策，强化普惠制培训，努力拓宽就业渠道，完成实名制就业10万人次，培训下岗失业人员2万人，四个城区的创业市场在6月底前竣工。

　　 确保社会大局稳定。以“争创全省综治工作先进市”为载体，构建以政法机关为主体、属地为基础、人防物防技防相结合的新型治安防控体系，加强城区电子监控网络建设，严厉打击各种违法犯罪活动，创建平安县区，切实增强群众安全感。按照“属地管理、分级负责，谁主管、谁负责”的原则，严格信访工作责任制，整顿和规范信访秩序，努力化解各种矛盾，逐年解决历史问题，去省、进京集体访大幅下降。

　　 全面加强安全监管。以对人民群众生命财产高度负责的精神，认真落实安全生产责任制，加大监管力度，严格责任追究，深入开展矿山、石油化工、建筑、交通、消防、危险化学品等行业领域的专项整治，全面排查和消除各类事故隐患，坚决遏制重特大事故的发生。加强食品药品安全监督管理，预防和控制各类突发公共卫生事件的发生。完善应急救援体系，提高对各类突发事件的反应和处置能力。

　　 强化和谐社区建设。建设和谐抚顺，基础在社区。进一步提升社区功能，强化社区作用，使之更好地在服务居民、维护治安、反映社情民意等方面发挥基础性作用，真正成为政府联系群众的桥梁和纽带。积极建设和谐邻里、和谐家庭、和谐人际关系，大力开展创建无集体访、无重大刑事案件、无失业的“三无”社区活动。

　　 努力为群众排忧解难。拓宽联系群众渠道，高度重视群众最关心、最直接的利益问题，建立健全为民办实事的长效机制，年年办好一批财力能承受、见效快、受益面广的实事。进一步发挥市长公开电话作用，提高对群众反映问题的解决率；完善资助体系，争取所有孩子不因贫困而失学，采取各种有效措施，确保不因贫困发生意外事件。加大水厂和供水设施改造力度，彻底解决居民吃原水问题；积极推进88条街巷路改造，解决居民出行难问题；深化热费改革，加强对供热单位监管，提高供热质量；新建、改造与开放沿街部分单位内部厕所相结合，解决市民如厕难。

　　(八)坚持“科教兴市”和“人才强市”战略

　　全面发展各项社会事业增强科技创新对经济的支撑作用。坚持引进消化和自主研发相结合，整合地方高校、科研院所和企业的科研力量，重点围绕资源深加工和提升传统产业开展技术攻关，全年完成科技成果135项，新增科技型企业40户，高新技术产业增加值增长25%。支持辽宁石油化工大学和石化研究院、煤炭研究院加快发展。

　　 加强人才和教育工作。树立人才资源是第一资源的理念，坚持用事业凝聚人才、用实践造就人才、用机制激励人才、用法制保障人才，不拘一格用人才。把教育放在优先发展地位，整合教育资源，全面提高教育教学质量；结合新一轮“普九”达标，调整优化中小学布局，撤并农村中小学64所，改造D级危房2万平方米；完成棚户区学校调整改造，新建、改建九年一贯制学校6所；大力发展职业教育，加强对在岗职工的培训，适应地区经济社会发展需要。

　　 全面发展其他各项社会事业。加强以学雷锋为主线的精神文明建设，坚持教育引导和典型示范相结合，提高公民的公德意识和社会文明程度，倡导科学、健康、文明的生活方式和城市新风。深入开展拥军优属活动，争取再创全国“双拥”模范城。积极开展健康向上、丰富多采的群众性文化活动，力争使“满族秧歌”进入国家级非物质文化遗产保护名录。扩大农村新型合作医疗范围，加强三级医疗预防保健网和农村中心卫生院及偏远地区的卫生院建设，社区卫生网络覆盖面达到100%。认真贯彻《全民健身计划纲要》，广泛开展全民健身活动，承办好辽宁省第六届少数民族运动会，争取在省十运会上取得好成绩。提高广播电视的节目和传输质量。切实维护妇女、儿童、老年人、残疾人的合法权益。稳定低生育水平，提高人口素质。加大社会事业闲置资产的盘活力度。

　　(九)加强自身建设，继续打造人民满意政府要超前谋划、推进发展

　　加快发展必须谋划先行。立足长远和全局，打破分工、行业和区域界限，搞好超前谋划，特别要抓好经济与社会发展，城市规划、建设与管理，财政资金，政府资产和政府负债等五大统筹，实现经济社会的协调发展和资源利用效率效益的最大化。切实增强忧患意识和责任意识，集中时间精力，紧抓项目不放。提高决策水平，对重大问题坚持做到没有经过调研论证不决策，没有多方案比选不决策，没有履行相关程序不决策，防止和避免决策失误。进一步规范经济秩序，营造统一、公平、有序的市场竞争环境。

　　 要狠抓落实、提高效率。振兴抚顺，重在落实。严格目标管理，层层落实责任，明确办事时限、标准和责任人，加强绩效考核。大力弘扬“棚改”和“抗洪”精神，坚持少说多做，崇尚实干创新，精简会议文件，坚决反对形式主义和弄虚作假。加强上下左右和内部之间的协调沟通和相互配合，最大限度地实现信息共享和形成合力。深化审批制度改革，加强政务公开和资源整合，完善公共行政服务中心功能，优化工作流程，精简办事程序；增强服务意识，继续开展“行风政风热线”和“双评”活动，建立投资者评价、舆论监督、组织考核相结合的考评体系，严肃查处破坏软环境和损害群众利益的案件，全力改善地区投资环境。

　　 要依法行政、廉洁自律。认真执行人大及其常委会的决议和决定，自觉接受人大的法律监督和政协的民主监督，提高人大代表建议和政协提案的办理质量；及时听取各民主党派、工商联、无党派人士和工会、共青团、妇联等群团组织的意见和建议，拓宽、畅通与社会各界的联系。坚持依法治市，搞好“五五”普法，认真贯彻《公务员法》和《全面推进依法行政实施纲要》，加强公务员队伍的能力和素质建设，落实行政执法责任制和执法过错责任追究制，提高依法行政水平。坚持不懈地开展廉政建设和反腐败斗争，建立健全教育、制度、监督并重的惩治和预防腐败体系，成立招投标中心，加强对项目竞标、产权交易、土地出让等重点领域的管理，从源头上铲除腐败滋生蔓延的土壤。

　　 各位代表，历史赋予抚顺难得机遇，时代为我们提供了广阔舞台。人民群众对政府的信任和支持，给了我们无穷的力量和必胜的信心。只要我们紧紧依靠全市人民，解放思想，夙兴夜寐，开拓进取，务实苦干，就一定能够全面完成今年的各项工作任务，“十一五”目标的实现、抚顺的转型振兴，就一定能够早日成为现实！抚顺的明天一定会更加美好！
